# Vaccine chronicle in Japan

**DOI:** 10.1007/s10156-013-0641-6

**Published:** 2013-07-09

**Authors:** Tetsuo Nakayama

**Affiliations:** Laboratory of Viral Infection I, Kitasato Institute for Life Sciences, Shirokane 5-9-1, Minato-ku, 108-8641 Tokyo

**Keywords:** Vaccine, Surveillance, MMR, DPT, Voluntary vaccines, Recommended vaccines

## Abstract

The concept of immunization was started in Japan in 1849 when Jenner’s cowpox vaccine seed was introduced, and the current immunization law was stipulated in 1948. There have been two turning points for amendments to the immunization law: the compensation remedy for vaccine-associated adverse events in 1976, and the concept of private vaccination in 1994. In 1992, the regional Court of Tokyo, not the Supreme Court, decided the governmental responsibility on vaccine-associated adverse events, which caused the stagnation of vaccine development. In 2010, many universal vaccines became available as the recommended vaccines, but several vaccines, including mumps, zoster, hepatitis B, and rota vaccines, are still voluntary vaccines, not universal routine applications. In this report, immunization strategies and vaccine development are reviewed for each vaccine item and future vaccine concerns are discussed.

## Dawn of vaccines in Japan

The dawn of vaccinology was the first scientific systematic investigation of the cowpox vaccination by Edward Jenner in 1796, although several variations in approach were performed using dried pus from smallpox skin lesions in Central Asia, China, and Turkey [1]. Jenner’s cowpox vaccination procedure was introduced into Japan in the Edo era by Philipp F.B. von Siebold. Sporadic nationwide outbreaks occurred at that time, which caused social, economic, and political stagnation, and doctors of herbal traditional medicine, studying Western modern medicine, wanted to use Jenner’s cowpox vaccine as a preventive procedure for smallpox. Many attempts were made to import the cowpox seed, but these did not succeed because Jenner’s cowpox vaccine is a live vaccine: it was inactivated during long-term transportation or if the inoculation chain in children was interrupted. It was finally introduced to Nagasaki in 1849, bringing the vaccination scar through the idea proposed by Dr. Souken Narabayashi, who was the chief doctor of Nabeshima-Han (Saga Prefecture). The vaccination procedure became available at the Shutousyo (Vaccination Institute) in Osaka and Edo cities, which was the origin of the School of Medicine of Osaka and Tokyo Universities [2]. Jenner’s cowpox vaccine gained in popularity because of its distinct effectiveness against smallpox. However, some opinions were against vaccination because of misunderstanding involving unreasonable and nonscientific rumors, as has recently been observed.

The Japanese government in the Meiji era decided that all Japanese people should be immunized with the vaccine for smallpox, which was stipulated in 1876, and a vaccination law against smallpox started in 1910. The present immunization law was implemented in 1948 under occupation by the United States (US). Issues related to vaccine development and immunization policies are summarized in Table 1. There have been two turning points for amendments to the immunization law: the compensation remedy for vaccine-associated adverse events in 1976, and the concept of private vaccination in 1994. These two turning points were attributed to vaccine-associated adverse events or accidents and lawsuits against serious neurological adverse events after immunization with vaccinia and the measles, mumps, and rubella-combined vaccine (MMR) [3]. In 1992, the regional Court of Tokyo, not the Supreme Court, set the governmental responsibility for vaccine-associated adverse events because the government did not make an effort to enlighten the public and doctors by explaining the possible adverse events associated with vaccinations, even though immunization was recommended to be compulsory [3]. This lack of information was a major reason why the government was reluctant to take active immunization strategic action, leading to the so-called long-term vaccine gap after the discontinuation, in 1993, of MMR, which had been introduced in 1989, because of the unexpectedly high incidence of aseptic meningitis caused by mumps vaccine components [4, 5]. The mechanisms behind the higher incidence of aseptic meningitis with the combined live MMR vaccine than with monovalent mumps vaccines were not clearly identified. Thereafter, new vaccines were not introduced, but many pediatric vaccines have been approved in developed countries, with the implementation of recommended vaccines, which shows that vaccine-preventable diseases should be controlled with available vaccines [6–9]. *Haemophilus influenzae* type b conjugated with tetanus toxoid (Hib) was introduced in 2008, and 7-valent *Streptococcus pneumoniae* conjugated vaccine with recombinant diphtheria toxoid (PCV7) and human papilloma virus vaccines (HPV) became available in 2010. Rotavirus vaccines were introduced in 2012. Several issues concerning vaccines in Japan are discussed in this article.

**Table 1:** History of immunization and vaccine development in Japan

1948: Immunization Law [Smallpox, Diphtheria, Typhoid fever, Salmonella Paratyphi, Pertussis, Tuberculosis, Typhus, Plague, Cholera, Scarlet fever, Influenza, Leptospirosis]	
1951: Preventive law against tuberculosis.	
1961: The polio vaccine was recommended.	
1962: School immunization with the influenza vaccine	Adverse events after the smallpox vaccination 1968–1970
1968: DPwT was recommended vaccination 1968–1970	
1976: Amendment of the immunization law for a compensation remedy for adverse events: Recommended obligatory [Smallpox, Diphtheria, Tetanus, Pertussis, Polio]; Temporarily [influenza, JEV]	DPT accidents 1974–1975
1977: The rubella vaccine was recommended.	
1978: The measles vaccine was recommended.	
1980: Eradication of smallpox and stopped being used.	
1981: The mumps vaccine was licensed.	MMR scandal 1989–1993
1985: The hepatitis B vaccine was licensed for the prevention of vertical transmission in1986.	
1994: Ammendment for private vaccination: Recommended [DPT, Polio, Measles, Rubella, JEV] Voluntary [influenza, VZV, Mumps]	
1995: The hepatitis A vaccine was licensed,	
2001: The influenza vaccine was recommended for the elderly >65 years.	
2005: BCG was recommended for infants 0–6 months of age.	JEV ADEM 2005
2005: The JEV vaccination was interrupted until 2009 and a booster at 14 years was stopped.	
2006: The two-dose schedule was started, using the MR combined vaccine.	
2009: Pandemic 2009 vaccines were imported from GSK and Novartis.	
2010: Hib, PCV7, and HPV were temporarily recommended.	

*DPwP* Whole cell pertussis vaccine combined with diphtheria and tetanus toxoids, *JEV* Japanese encephalitis virus vaccine, *MMR* Measles, mumps and rubella-combined vaccine, *VZV* Varicella zoster virus vaccine, *ADEM* Acute disseminated encephalomyelitis, *Hib* Haemophilus influenzae type b vaccine, *PCV7* 7-valent Str. pneumoniae vaccine, *HPV* Human Papilloma virus vaccine

## Immunization law and schedule

The Japanese immunization law is complicated with double-standard categories: routine recommended and voluntary vaccination. Routine recommended vaccines consist of BCG, acellular pertussis vaccine (DTaP), measles and rubella combined vaccine (MR), inactivated polio (IPV), Hib, PCV7, HPV, and Japanese encephalitis vaccine (JEV). Voluntary vaccines are hepatitis B (HBV), mumps, varicella, and rotavirus vaccines. The difference between the two is the cost of immunization; routine recommended vaccines are principally covered by the regional government [10, 11]. Until 1994, immunization was performed by mass vaccination in regional Public Health Centers. It was replaced by private vaccination, derived from the concept that it is better that vaccinations are performed by children’s family doctors who are familiar with their health conditions. Although this concept was easily accepted by general physicians, mass vaccination of BCG still continued in some regions.

**Fig. 1:**
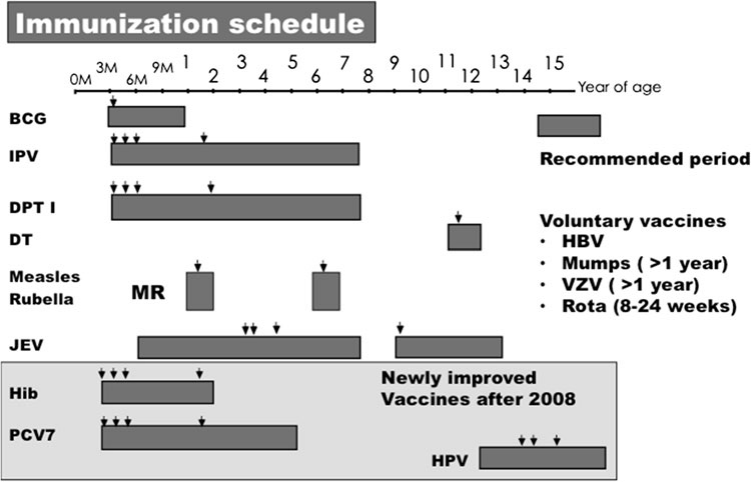
Immunization schedule. BCG, IPV, DPT, DT, MR, JEV, Hib, PCV7, and HPV were recommended vaccines and HBV, Mumps, VZV, and Rota vaccines were voluntary vaccines. *Arrows* show the recommended timing for vaccinations. *BCG* Bacillus Calmette Guérin, *IPV* Inactivated polio vaccine, *DPT* Diphtheria and tetanus toxoids combined with pertussis vaccine, *DT* Diphtheria and tetanus toxoids, *MR* Measles and rubella-combined vaccine, *JEV* Japanese encephalitis vaccine, *Hib* Haemophilus influenzae type b vaccine, *PCV7* 7-valent Str. pneumoniae vaccine, *HPV* Human papilloma virus vaccine, *HBV* Hepatitis B virus vaccine, *VZV* Varicella zoster virus vaccine

In 2010, Hib, PCV7, and HPV began to be used as temporarily recommended vaccines, and the cost was partially supported by the regional governments [12]. Vaccination coverage of routine recommended vaccines is more than 90–95 % for BCG, DTaP, OPV, and MR and 80 % for JEV, whereas that of voluntary vaccines is less than 30–40 %. During 1990–2000 polyvalent combined vaccines were developed in the EU and widely used. There is no licensed polyvalent vaccine in Japan, and the vaccination schedule became much tighter than that in the 1990s, especially in very young infants less than 6 months of age (Fig. 1). Simultaneous administration of several vaccines was recommended by the Japanese Pediatric Association, as has been conducted in the US and EU [3, 4]. In March 2011, seven infants died within a week of receiving DTaP, Hib, PCV7, or BCG. The newly introduced Hib and PCV7 were temporally discontinued, but were restarted 1 month later because the risk of serious adverse events was not higher than that reported in developed countries. Simultaneous administration has been safely and effectively performed in the US and EU; however, the incidence of serious adverse events has been reported as 0.02–1 in 100,000 [13]. Therefore, simultaneous immunization is now performed without a high level of confidence. Careful surveillance monitoring and scientific investigations are required to define the safety of simultaneous immunization.

## Measles and rubella elimination

**Fig. 2:**
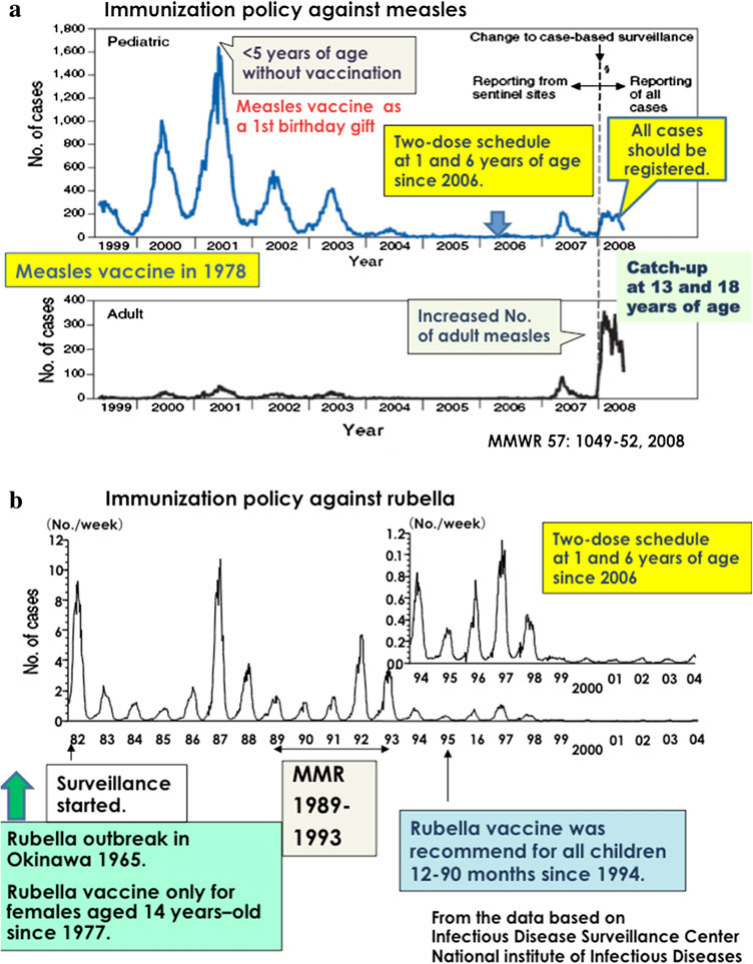
Surveillance results of measles (**a**) and rubella (**b**), and the changes in immunization policies. Measles and rubella vaccines were recommended in 1978 and 1977, respectively. The MMR vaccine was used between 1989 and 1993, and the target generation of the rubella vaccine shifted from 14-year-old female schoolchildren to all infants 12–90 months of age. The two-dose schedule of the MR combined vaccine started in 2006. A catch-up campaign started in 2008 for an additional 5-year schedule for children 13 and 18 years of age. *MMR* measles, mumps, and rubella-combined vaccine

In Japan, live attenuated measles vaccines were developed in the 1970s, and four strains were licensed (three strains are used at present) with the implementation of recommended immunization in 1978 [14]. Five strains of live attenuated rubella vaccines (three strains are used at present) were developed and recommended for female students aged 14 years in 1977 [15]. Surveillance data and changes in the vaccination policy against measles and rubella are shown in Fig. 2. The MMR vaccine was used between 1989 and 1993 but was discontinued in 1993. Measles and rubella monovalent vaccines have been used for children aged 12–90 months since 1994 to control measles and rubella because the number of patients with congenital rubella syndrome did not decrease as a result of the vaccination of only young females since 1977.

Regarding the reporting system for measles in Japan, through 3,000 sentinel clinics or hospitals for pediatric infectious diseases and 450 clinics for adult measles surveillance, patients with clinically suspected measles were reported to Regional Health Care Centers. In the late 1990s to early 2000s, 20,000–30,000 cases of measles, including several dozen deaths, were reported yearly. A total of 2,034 cases of measles, including 8 deaths, were reported in a severe measles outbreak in Okinawa in 1998–1999 [16]. Many of the deaths occurred in infants under 1 year of age. A large measles outbreak was observed in 2001 in Japan. Among 33,812 reported cases, most patients were under 5 years of age and had not been vaccinated. Through a vaccination campaign to increase immunization coverage at 1 year of age, the number of reported cases decreased to 545 in 2005. The Japanese Government implemented a two-dose strategy in 2006, a combined measles and rubella vaccine (MR) for children at 1 and 6 years of age [17]. Therefore, the elimination of measles was expected. However, patients with measles were increasingly reported in March 2007, and this outbreak subsequently expanded throughout the Japanese districts, peaking in the middle of May. Furthermore, several reports indicated measles transmission by Japanese travelers or participants in an international sporting event [18–20]. This outbreak showed different characteristics, demonstrating that most patients were young adults or adolescents attending high school and university students, with a much lower proportion of young infants, at the early stage of the outbreak [21]. Cases of measles were reported in all age populations, with a total of 3,105 pediatric cases and 959 adult patients being reported in 2007. The number of patients with measles was the highest between 1 and 4 years of age, accounting for 40–50 % in 2001, which decreased to 22 % in the outbreak of 2007. A significant shift in the age distribution of cases of measles in 2007 was observed to be 10–14 years or older, accounting for 44 % in 2007 [22].

To reduce the number of patients with measles, an additional MR catch-up campaign was started for teenagers at the age of 13 and 18 years (MR III and IV) from 2008 for a 5-year schedule. After 2008, all cases with measles had to be registered, and the number of patients with measles was reduced to 457 cases in 2010 (3.58 cases per million), with some imported genotypes [23]. In 2011, measles was introduced from the EU by a journalist who was collecting the news of the earthquake, tsunami, and nuclear power disaster, and a total of 442 patients with measles were finally reported [24]. In 2012, 293 patients were reported, just on the edge of measles elimination of 1 case in 1,000,000, and most cases were identified as importations from Southeast Asia and the EU [25].

Global measles vaccination coverage increased from 72 % in 2000 to 82 % for the first dose in 2007, and the two-dose immunization strategy was recommended for countries with high coverage of the first-dose measles vaccine, at more than 95 %. Most countries (88 %) now implement the two-dose strategy [26]. However, measles transmission has increased, and outbreaks have become widespread since late 2009 in the EU region because of the failure to immunize susceptible populations [24]. The World Health Assembly updated the goal of measles elimination to a 95 % reduction in measles mortality by 2015, compared to 2000 [27].

The rubella vaccine strategy was markedly changed in 1994. Before 1989, the rubella vaccine was administered to 14-year-old girls, but the vaccine target has changed to all children aged 12–90 months. Boys more than 90 months of age and girls from 90 months to 14 years of age were not enrolled as immunization targets in the transition period. Even though a temporal catch-up campaign was conducted to cover the immunization gap, vaccine coverage was extremely low [15, 28]. According to the immunization gap in younger generations around 30 years of age, an outbreak of rubella was observed in 2011–2013, with some imported cases from Southeast Asia, resulting in congenital rubella syndrome [29]. Rubella is now prevalent (in 2013) among men around 30 years of age who have not been immunized because of the immunization gap. Through the enhanced network activity of measles and rubella elimination, the elimination of rubella has been targeted in accordance with measles elimination, using the measles and rubella combined vaccine [30, 31].

## Replacement of oral polio vaccine (OPV) with inactivated polio vaccine (IPV)

**Fig. 3:**
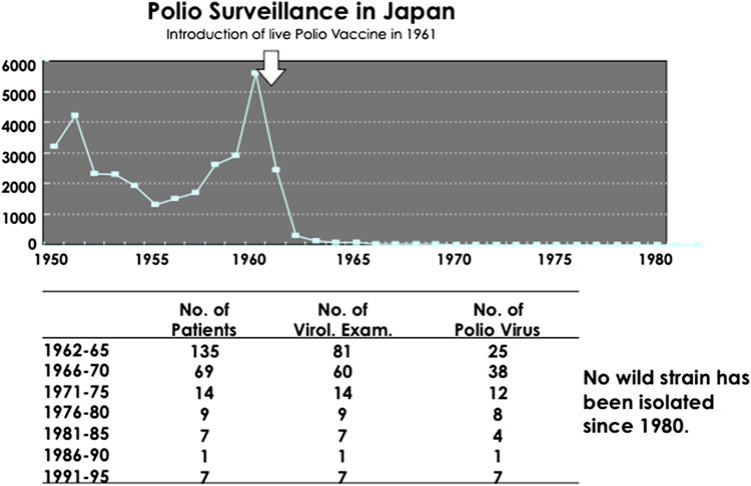
Polio surveillance in Japan since 1950. A peak number of patients with polio was observed in 1960, and the live polio vaccine was introduced in 1961 (*upper panel*). After 1962, the number of patients with polio decreased, and no wild strain has been isolated since 1980

Surveillance data of reported cases of polio paralysis are shown in Fig. 3. In 1960, a nationwide outbreak was observed, and approximately 5,800 patients with paralytic polio were reported. A similar level of outbreak seemed to be observed in 1961, and the Japanese government decided to import sufficient doses of OPV for all Japanese children. Within a month, 15 million doses were given to all Japanese children less than 5 years old. Around 1960, although IPV was under investigation and a clinical trial of imported OPV was also underway in Japan, the importation of OPV was politically decided. After the introduction of OPV in 1961 and afterward, the number of polio cases decreased [32]. After 1980, no wild strain was isolated from patients suspected of flaccid paralytic polio. All cases of paralytic polio were identified as vaccine-associated paralytic polio (VAP). The incidence of VAP was recently shown to be one in 1.4 million recipients in Japan. Clinical trials of domestic IPV produced from Sabin’s live oral polio vaccine strains were performed beginning in 1998, but the application was withdrawn. Considering the practical way of immunization, the development of IPV combined with DTaP was more desirable than IPV alone. OPV was replaced with IPV in most developed countries, but it was delayed by the standstill of the IPV trial in Japan. Some guardians and pediatricians could not wait for the licensure of domestic DTaP/IPV and imported the IPV vaccine privately at their own responsibility. In 2012, IPV was allowed for use as a recommended vaccine imported from Sanofi and domestic DTaP/IPV vaccines [33]. The wild poliovirus was imported in several situations from countries where wild polio has been circulating, and the high levels of vaccine coverage have been maintained. In addition to disease surveillance, environment surveillance of the vaccine for polio virus should focus on sewage monitoring [34].

## Is the DTaP vaccine effective in controlling pertussis?

**Fig. 4:**
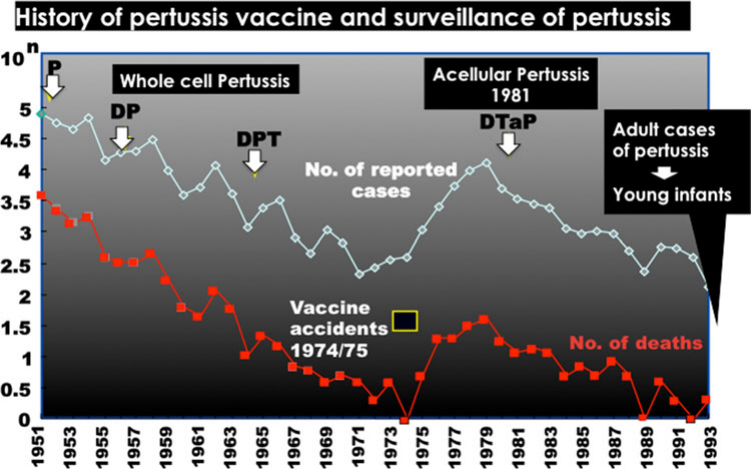
History of the pertussis vaccine and surveillance of the number of reported cases of pertussis and pertussis deaths. The DPT vaccine was recommend in 1968. *P* Pertussis vaccine, *DP* Diphtheria toxoid combined with pertussis vaccine, *DPT* Diphtheria and tetanus toxoids combined with pertussis vaccine, *DTaP* acellular pertussis vaccine combined with diphtheria and tetanus toxoids

Pertussis is still a serious illness in young infants, and causes whooping cough, apnea, cyanosis, choking, and encephalopathy [35]. In Japan, the whole-cell pertussis vaccine was developed in 1949 and was combined with diphtheria and tetanus toxoids (DTwP). The results of pertussis surveillance and changes in vaccine strategy are shown in Fig. 4. Although febrile adverse illness was observed in 10 % of the recipients of DTwP, with local reactions of redness at 50–60 % and induration at 20 %, this vaccine was accepted. A routine immunization schedule was implemented with DTwP in 1968, resulting in a reduction in the reported cases of pertussis and pertussis deaths. In 1974–1975, two accidental deaths were reported after the administration of DTwP and, thereafter, DTwP was temporarily discontinued. It was reintroduced for children aged 2 years old and older, or the DT vaccine was used instead of DTwP. The number of pertussis patients and pertussis deaths increased because of the low vaccine coverage [36, 37].

In 1981, a new type of acellular pertussis was developed, and a combined vaccine (DTaP) was introduced into recommended immunization practice. Principally, two types of DTaP vaccine (Biken-type, B-type; Takeda-type, T-type) were developed: the B-type consisted of two major antigens, pertussis toxin (PT) and filamentous hemagglutinin (FHA), and the T-type contained pertactin and fimbriae in addition to PT and FHA [38, 39]. Nationwide monitoring of clinical adverse events demonstrated low reactogenicity and sufficient antibody responses similar to natural infection. Since 1981, the number of pertussis patients has decreased after the acceptance of DTaP. However, the incidence of pertussis has recently been increasing in adults since 2002 in Japan, and several outbreaks on university campuses and in high schools and offices have been reported [40, 41]. Adult patients of pertussis are difficult to diagnose because of nontypical clinical features, including a prolonged cough. Also, the isolation of *Bordetella* or detection of the pertussis genome is not always successful because of the short duration of excretion of *Bordetella* influenced by the empirical administration of antibiotics or vaccination history [41, 42]. A surveillance system is currently under construction in Japan, based on a genetic diagnosis by loop-mediated isothermal amplification (LAMP) for detection of the pertussis genome [43].

DTaP was adopted by global vaccine makers because of the lower incidence of adverse events than that with DTwP, and multivalent combined vaccines, such as DTaP/Hib/IPV/HBV hexavalent vaccines, were developed based on DTaP. Pertussis is principally an infectious children’s illness causing whooping and prolonged cough, and the Advisory Committee on Immunization Practices (ACIP) recommended a five-dose DTaP schedule, at ages 2, 4, 6, 15–18 months, and 4–6 years, instead of the previous DTwP in the US in 1997 [44]. In the 1990s, the incidence of pertussis at an older age increased in many countries because of waning immunity after primary childhood immunization and antigenic changes in pertussis, and adolescent pertussis was identified as the source of the transmission of pertussis to young infants through enhanced surveillance studies [45]. In Japan, the number of newborn pertussis cases increased in household contact [46]. In 2005, the tetanus toxoid, combined with a reduced concentration of diphtheria toxoid and acellular pertussis components (Tdap) vaccine, was licensed in the US, and the ACIP recommended that adolescents aged 11–18 years old should receive a single dose of Tdap for a booster immunization [47]. It is now recommended for all generations from 19 to more than 64 years of age who have not been vaccinated in the past 10 years [48]. In Japan, DT was recommended at the age of 11–12 years, and the lack of pertussis booster immunization is one of the reasons why the number of patients with pertussis has increased in adults. The booster effect of a reduced volume of DTaP was investigated instead of the DT vaccine at 11–12 years of age, and 0.2 ml DTaP induced sufficient antibodies against PT and FHA without serious adverse events [49]. Even with high vaccine coverage, the number of pertussis patients increased globally because of the short duration of vaccine immunity. Several DTaP candidates containing additional protective antigen(s) are now under investigation [50].

## Does the influenza split vaccine prevent infection?

**Fig. 5:**
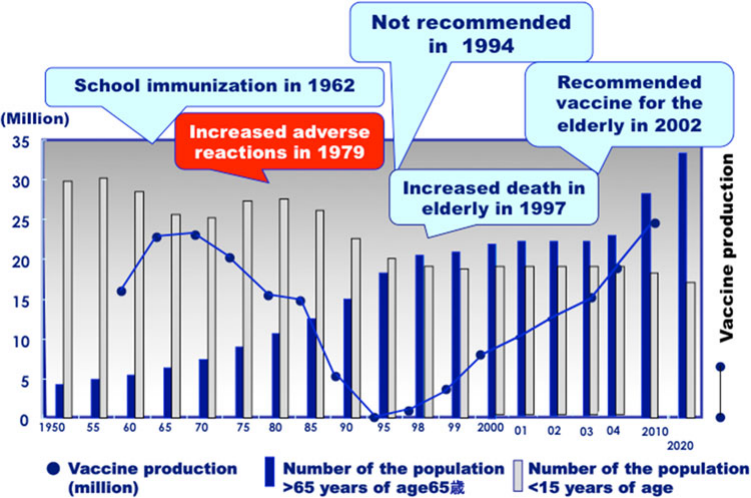
Changes in the immunization strategy of the influenza vaccine, population more than 65 years and less than 15 years of age, and vaccine production in million doses

Two types of influenza virus vaccines are now globally available, inactivated and cold-adapted live attenuated vaccines. There are three types of inactivated vaccines: whole virion, split, and subunit inactivated vaccines. The whole virion inactivated vaccine induced febrile reactions after the vaccination, and thereafter the split vaccine was licensed in 1972 in Japan, which has been used for more than 40 years with a lower incidence of febrile reactions. The split vaccine is made by destroying the structure of virus using detergents and ether to remove their lipid components from the formalin-inactivated whole virion. The HA subunit vaccine is purified from the HA fragments zone [51]. Changes in immunization policies, vaccine production, and the population aged less than 15 and more than 65 years are shown in Fig. 5. The transmission of influenza was believed to be associated with contact with schoolchildren, and, thereafter, the influenza vaccine has been recommended every year as school immunization in primary schools since 1962 [52]. In the 1960s, the pediatric population (<15 years of age) was more than 20 million, and more than 25 million doses of influenza vaccine were produced. The effects of school immunization on decreasing the social impact of influenza were questionable, and a comparative study was performed. There was no difference in the number of reported cases, number of hospital visits, and cost of healthcare insurance among several cities with or without school immunization in Gunma Prefecture in the early 1980s. This study provided evidence that school immunization had no effect on reducing the impact of influenza in the community, but had a limited effect on an individual basis [53, 54]. The influenza vaccine strategy was shifted from an obligatory routine vaccine to a voluntary vaccine in 1994. School immunization was interrupted in 1995, and the total amount of vaccine produced was at its lowest, 0.35 million doses. A large outbreak of H3N2 was observed in 1997, and several deaths were reported in many nursing homes for the elderly as social topics. It has been recommended as a routine recommended vaccine for the elderly more than 65 years of age since 2002 for the benefits of vaccine recipients [55].

Three pandemics of influenza occurred in the 20th century. The most devastating pandemic dated back to 1918 and was known as Spanish flu. It was caused by a highly pathogenic H1N1 influenza virus transmitted through some animals from an avian pathogenic virus and is estimated to have killed 40–50 million people [56]. In 1957, Asian influenza A/H2N2 caused the second pandemic, and Hong Kong influenza A/H3N2 appeared as the third pandemic in 1968. Seasonal influenza outbreaks or epidemics are caused by an antigenic drift of A/H1N1 or A/H3N2, whereas these pandemics appeared as an antigenic shift, leading to a new strain, which is thought to be a re-assortment with the non-preexisting features of hemagglutinin (HA) or neuraminidase (NA) in human influenza viruses. After the 1968 pandemic of A/H3N2, several cases and small local outbreaks were reported, caused by new strains, H5, H7, or H9, and were considered to be from poultry, with H5 being very close to humans as a target for vaccine development [57]. A regional outbreak of H5 was reported in Hong Kong in 1997, and 6 of 18 patients died, causing an H5 pandemic threat [58]. Sporadic H5 transmission on poultry farms and in migratory birds has spread across Asia to the EU and Africa, and approximately 610 cases of human H5 infection have been reported at present in 2013 since 2003, showing a high mortality rate of approximately 60 %. Most cases have involved close and direct contact with poultry, with no definite evidence of human-to-human transmission. There are several barriers to human-to-human transmission: receptor usage of the HA protein, cleavage efficiency by cellular protease, and host factors. H5N1 is considered to be a target for the pandemic vaccine, and the WHO addressed sharing viruses and sequence information for a future pandemic vaccine development [57, 59]. The development of an effective and safe vaccine is expected to mitigate the threat of a pandemic.

Several types of H5 vaccines have been developed, basically based upon the HA split, subunit vaccine, or whole virion inactivated with adjuvant. In Japan, alum-adjuvanted H5N1 whole virion inactivated vaccine (WIV) (alum concentration, 300 μg/ml) was developed using a genetically engineered reassortant, the NIBRG-14 strain, originating from H5N1/A/Vietnam/1194/2004. In a clinical phase II/III trial in healthy adults, alum-adjuvanted WIV (HA protein, 15 μg) led to favorable immunogenicity, >70 % sero-conversion rate in neutralization tests (NT) antibodies, without causing any serious systemic illnesses [60]. However, when it was administered to young infants and children at a reduced dose, 7.5 or 3 μg, a high body temperature (≥38.0 °C) was observed in approximately 60 % of recipients less than 7 years of age, and, unexpectedly, NT antibody titers were higher in children than in the clinical trial in adults. These phenomena were associated with the enhanced production of inflammatory cytokines [61].

## Introduction of Hib, PCV7, and HPV

Hib and PCV are the major pathogens of bacterial meningitis and invasive systemic bacteremia, and they cause serious pneumonia. In the past, bacterial infection was believed to be treatable with antibiotics through early diagnosis and was not a target for vaccine development before 2000 in Japan. However, a shift led to the development of vaccines in the late 1980s in the US. The appearance of resistant strains provided an impetus for the introduction of vaccines. In Japan, the surveillance study of the incidence of Hib meningitis was conducted, which showed the incidence was 8.3 per 100,000 children less than 5 years of age [62, 63]. These surveillance results estimated 600 cases of serious invasive Hib infection, and then, the Hib vaccine was introduced. In the postmarketing study, the practical usage of Hib simultaneously administered with DPT was confirmed to be safe and effective, similar to separate administration [64]. It was allowed in 2008, and Hib was the first vaccine imported from a foreign country. Thereafter, PCV7, HPV, and Rota vaccines were licensed. Hib, PCV7, and HPV vaccines were temporarily adopted as routine recommended vaccines in 2010 with tentative financial support and were engaged to be covered as routine recommended vaccines in 2013 [10]. After the introduction of Hib and PCV7, the incidence of serious invasive infection decreased whereas the *Streptococcus pneumoniae* 6B and 19A serotypes uncovered by PCV7 are increasing, with a higher number of penicillin-resistant strains [65, 66]. Hib infection became controlled but *S. pneumoniae* has approximately 100 serotypes, using serotype replacement after the introduction of PCV7 and PCV13 to be licensed.

## Action for the routine immunization of mumps, zoster, and hepatitis B vaccines

Five live mumps vaccine strains were developed in the 1970s from domestic wild strains isolated in the 1960s and 1970s [67, 68]. MMR vaccines containing four domestic vaccine strains were used, but were discontinued because of the unexpected high incidence of aseptic meningitis. Thereafter, monovalent mumps vaccines were used and the incidence of aseptic meningitis was evaluated. We reported that the incidence of aseptic meningitis was 13/1,051 (1.24 %) in patients with symptomatic natural mumps infection and was estimated to be 0.7–1.1 % of overall infections considering asymptomatic infections, and 10/21,465 (0.05 %) in vaccine recipients [69]. Although aseptic meningitis is considered to be an apparent adverse event of the mumps vaccine, its incidence is considerably lower than among those with symptomatic natural infections. It provides informative findings for consideration of resuming the mumps vaccine as a part of a routine immunization schedule for Japanese children. Regarding mumps deafness, the incidence of deafness was previously believed to be 1 in 15,000 [70], but irreversible mumps deafness occurred at a higher incidence, in 1 case per 1,000 [71]. Mumps deafness is one of the targets for vaccine implementation. Mumps outbreaks were observed every 3–5 years because of low vaccine coverage, less than 40 %, because the vaccine was voluntary.

The varicella zoster virus vaccine OKA strain was developed in Japan in 1974, and is the only strain available in the world [72]. Initially, it was developed for immunocompromised hosts who develop serious complications with chickenpox [73]. It causes no serious adverse reaction and protects against the onset of illness by immediate inoculation within 3 days of contact with patients in pediatric wards [74]. It was allowed for use in healthy infants, but the yearly epidemiological pattern did not change because of the low vaccine coverage, less than 40 % [75].

Mumps and zoster vaccines were universal vaccines in the US and EU but were voluntary in Japan [10, 12, 75]. The hepatitis B vaccine (HBV) is still a voluntary vaccine, as HBV was developed to interrupt the carrier through vertical transmission from carrier mothers positive for the HBe antigen [76]. HBV was given at 2, 3, and 5 months of age, and the number of carriers became markedly reduced. Recently, cases of nosocomial infections or horizontal transmission cannot be neglected, and HBV should be adopted as a universal vaccination [77]. Mumps, zoster, and HBV are still voluntary vaccines in Japan although they are globally recommended as universal vaccines. These vaccines are anticipated to be routine recommended vaccines.

## Requirement for future immunization

The disease surveillance system in Japan is based on 3,000 sentinel clinics or hospitals for pediatric infectious diseases and reflects the tendency of infectious diseases, not population-based incidences. The immunization strategy is decided based upon disease surveillance, and monitoring of vaccine-associated adverse events is important to assess the safety. It is now based on postmarketing surveillance in Japan and should be developed in a systematic administrative form, together with laboratory investigations. It is difficult to identify the relationship of vaccination to serious adverse events occurring within a few weeks after immunization, and, in most cases, a direct relationship could not be identified. In 2005, a serious case of acute disseminated encephalomyelitis (ADEM) was reported after vaccination with the Japanese encephalitis vaccine (JEV). At that time, JEV was produced from purified virus particles from mouse brains infected with Japanese encephalitis virus. Therefore, JEV has the potential to cause allergic encephalomyelitis. JEV was suddenly discontinued in a shortsighted political decision, without considering the effects of blank periods without JEV. At that time, tissue-culture JEV was ready to be marketed. Comprehensive decisions are required and should be made after scientific discussion.

No organization for decision making on immunization is currently systematized in Japan, such as the Advisory Committee on Immunization Practices (ACIP) of the US [12]. An investigational Committee on Immunization was organized to propose immunization strategies to decision makers and to discuss problematic issues based on the scientific evidence. However, this committee has been organized in the administrative agency, the Ministry of Health, Labor, and Welfare. Although issues on immunization should be discussed based on scientific evidence as a third party, it belongs to the political side at present. It may be hard to listen to the clinical needs of general physicians for the improvement of immunization practice. It should be organized for the purpose of promoting public health with a longitudinal vision for immunization policies and prompt responses to the critical issues, without the influence by political changes.

## Abbreviations

ACIP: Advisory Committee on Immunization Practices
BCG: Bacillus Calmette–Guérin
DTaP: Acellular pertussis vaccine combined with diphtheria and tetanus toxoids
DTwP: Whole cell pertussis vaccine combined with diphtheria and tetanus toxoids
FHA: Filamentous hemagglutinin
HA: Hemagglutinin
HBV: Hepatitis B virus vaccine
Hib: *Haemophilus influenzae* type b conjugated with tetanus toxoid
HPV: Human papilloma virus vaccine
JEV: Japanese encephalitis vaccine
IPV: Inactivated polio vaccine
LAMP: Loop-mediated isothermal amplification
MMR: Measles, mumps, and rubella-combined vaccine
MR: Measles and rubella-combined vaccine
NA: Neuraminidase
NT: Neutralization test
OPV: Live oral polio vaccine
PCV7: 7-valent *Streptococcus pneumoniae* conjugated vaccine with recombinant diphtheria toxoid
PT: Pertussis toxin
Tdap: Tetanus toxoid combined with a reduced concentration of diphtheria toxoid and acellular pertussis
VAP: Vaccine-associated paralytic polio
VZV: Varicella zoster virus vaccine

## References

[1] Plotkin SL, Plotkin SA, Plotkin S, Orenstein WA (2007). A short history of vaccination. Vaccines.

[2] Ogawa T. Igaku no rekishi. Chukoushinsho 39, Chuoukouronnsya (in Japanese); 1964.

[3] Kamiya H, Kamiya H. Learning from the US immunization administration. Nihon Rinsho. 2008; 66:61858–64 (in Japanese). 18939482

[4] Kimura M, Kuno-Sakai H, Yamazaki S (1996). Adverse events associated with MMR vaccines in Japan. Acta Paediatr Jpn.

[5] Ueda K, Miyazaki C, Hidaka Y, Okada K, Kusuhara K, Kadoya R (1995). Aseptic meningitis caused by measles-mumps-rubella vaccine in Japan. Lancet.

[6] CDC. Global routine vaccination coverage, 2010. MMWR. 2011; 60:1520–1522. 22071590

[7] Philippe D, Jean-Marie OB, Marta GD, Thomas C (2009). Global immunization: status, progress, challenges and future. BMC Int Health Hum Rights.

[8] Dennehy PH (2001). Active immunization in the United States: developments over the past decade. Clin Microbiol Rev.

[9] Plotkin SA (2009). Minireview: Vaccines: the fourth century. Clin Vaccine Immunol.

[10] Saitoh A, Okabe N (2012). Current issues with the immunization program in Japan: can we fill the “vaccine gap”?. Vaccine.

[11] Shimazawa R, Ikeda M (2012). The vaccine gap between Japan and the UK. Health Policy.

[12] Kamiya H, Okabe N (2009). Leadership in immunization: the relevance to Japan of the U.S.A. experience of the Centers for Disease Control and prevention (CDC) and the Advisory Committee on Immunization Practices (ACIP). Vaccine.

[13] Zepp F, Schmitt H-J, Cleerbout J, Verstraeten T, Schuerman L, Jacquet J-M (2009). Review of 8 years of experience with Infanrix hexa™ (DTPa-HBV-IPV/Hib hexavalent vaccine). Expert Rev Vaccines.

[14] Hirayama M (1983). Measles vaccine in Japan. Rev Infect Dis.

[15] Ueda K (2009). Development of the rubella vaccine and vaccination strategy in Japan. Vaccine.

[16] Nakayama T, Zhou J, Fujino M (2003). Current status of measles in Japan. J Infect Chemother.

[17] CDC: Progress toward measles elimination: Japan, 1999–2008. MMWR. 2008;57:1049–52. 18818586

[18] CDC. Multistate measles outbreak associated with an international youth sporting event—Pennsylvania, Michigan, and Texas, August–September 2007. MMWR. 2008;57:169–73. 18288074

[19] CDC. Outbreak of measles: San Diego, California, January–February 2008. MMWR. 2008;57:203–6. 18305451

[20] Delaportel E, Wyler CA, Sudre P. Outbreak of measles in Geneva, Switzerland, March–April 2007. Euro Surveill 2007;12:19. 10.2807/esw.12.19.03190-en 17868601

[21] Nagai M, Xin JY, Yoshida N, Miyata A, Fujino M, Ihara T (2009). Modified adult measles in outbreak in Japan, 2007–08. J Med Virol.

[22] National Institute of Infectious Diseases and Tuberculosis and Infectious Diseases Control Division, Ministry of Health, Labour and Welfare (2007). Measles and rubella in Japan, as of March 2006. IASR.

[23] Saito M, Takeda M, Gotoh K, Takeuchi F, Sekizuka T, Kuroda M (2012). Molecular evolution of hemagglutinin (h) gene in measles virus genotypes d3, d5, d9, and h1. PLoS ONE.

[24] CDC: Increased transmission and outbreaks of measles—European region, 2011. MMWR. 2011;60:1605–10. 22129994

[25] WHO: Measles virus nomenclature update: 2012. Wkly Epidemiol Rec 2012;87:73–81. 22462199

[26] CDC: Progress in global measles control and mortality reduction, 2000–2006. MMWR. 2007;56:1237–1241. 18046301

[27] WHO. Measles vaccines: WHO position paper. Wkly Epidemiol Rec. 2009;84:349–360. 19714924

[28] Terada K (2003). Rubella and congenital rubella syndrome in Japan: epidemiological problems. Jpn J Infect Dis.

[29] Tran DN, Pham NT, Tran TT, Khamrin P, Thongprachum A, Komase K (2012). Phylogenetic analysis of rubella viruses in Vietnam during 2009–2010. J Med Virol.

[30] CDC. Recommendations from Ad Hoc Meeting of the WHO measles and rubella laboratory network (LabNet) on use of alternative diagnostic samples for measles and rubella surveillance. MMWR. 2008;57:657–660. 18566565

[31] Goodson JL, Chu SY, Rota PA, Moss WJ, Featherstone DA, Vijayaraghavan M (2012). Research priorities for global measles and rubella control and eradication. Vaccine.

[32] Takatsu T, Tagaya I, Hirayama M, on behalf of the Poliomyelitis Surveillance Committee of Japan (1973). Poliomyelitis in Japan during the period 1962–68 after the introduction of mass vaccination with Sabin vaccine. Bull World Health Org.

[33] Scimizu H. Poliovirus vaccine. Uirusu. 2012;62:57–65 (In Japanese). 10.2222/jsv.62.57 23189825

[34] Miyamura T (2012). Ten years after polio eradication from the WPRO region: current status and future problems. Vaccine.

[35] Edwards KM, Decker MD, Plotkin SA, Orenstein WA, Offit PA (2008). Pertussis vaccines. Vaccines.

[36] Kimura M, Kuno-Sakai H (1988). Pertussis vaccines in Japan. Acta Paediatr Jpn.

[37] Kimura M, Kuno-Sakai H (1990). Development in pertussis immunization in Japan. Lancet.

[38] Sato Y, Kimura M, Fukumi H (1984). Development of a pertussis component vaccine in Japan. Lancet.

[39] Kuno-Sakai H, Kimura M, Watanabe H (2004). Verification of components of acellular pertussis vaccines that have been distributed solely, been in routine use for the last two decades and contributed greatly to control of pertussis in Japan. Biologicals.

[40] Otsuka N, Han HJ, Toyoizumi-Ajisaka H, Nakamura Y, Arakawa Y, Shibayama K (2012). Prevalence and genetic characterization of pertactin-deficient *Bordetalla pertussis* in Japan. PLoS ONE.

[41] Miyashita N, Kawai Y, Yamaguchi T, Ouchi K, Kurose K, Oka M (2011). Outbreak of pertussis in a university laboratory. Intern Med.

[42] Miyashita N, Kawai Y, Yamaguchi T, Ouchi K (2011). Evaluation of serological tests for diagnosis of *Bordetella pertussis* infection in adolescents and adults. Respirology.

[43] Kamachi K, Toyoizumi-Ajisaka H, Toda K, Soeung SC, Sarath S, Nareth Y (2006). Development and evaluation of a loop-mediated isothermal amplification method for rapid diagnosis of *Bordetella pertussis* infection. J Clin Microbiol.

[44] CDC. Recommendations and reports. Pertussis vaccination: use of acellular pertussis vaccines among infants and young children: Recommendation of the Advisory Committee on Immunization Practices (ACIP). MMWR. 1997;46:1–25. 9091780

[45] Wendelboe AM, Njamkepo E, Bourillon A, Floret DD, Gaudelus J, Gerber M, et al; Infant Pertussis Study Group. Transmission of *Bordetella pertussis* to young infants. Pediatr Infect Dis J 2007;26:293–299. 10.1097/01.inf.0000258699.64164.6d 17414390

[46] Nakamura A, Sakano T, Nakayama T, Shimonda H, Okada Y, Hanayama R (2009). Neonatal pertussis presenting as acute bronchiolitis: direct detection of the *Bordetella pertussis* genome using loop-mediated isothermal amplification. Eur J Pediatr.

[47] CDC. Preventing tetanus, diphtheria, and pertussis among adolescents: use of tetanus toxoid, reduced diphtheria toxoid and acellular pertussis vaccines. Recommendations of the Advisory Committee of Immunization Practices (ACIP). MMWR. 2006;55:1–34. 16557217

[48] CDC. FDA approval of expanded age indication for a tetanus toxoid, reduced diphtheria toxoid and acellular pertussis vaccine. MMWR. 2011;60:1279–11280. 21937974

[49] Okada K, Komiya T, Yamamoto A, Takahashi M, Kamachi K, Nakano T (2010). Safe and effective booster immunization using DTaP in teenagers. Vaccine.

[50] van der Ark AA, Hozbor DF, Boog CJ, Metz B, van den Dobbelsteen GP, van Els CA (2012). Resurgence of pertussis calls for re-evaluation of pertussis animal models. Expert Rev Vaccines.

[51] Wood JM, Williams MS (1998) In: Nicholson KG, Webster RG, Hay AJ (eds) Textbook of influenza. Blackwell, Oxford, pp 317–323

[52] Kawai S, Nanri S, Ban E, Inokuchi M, Tanaka T, Tokumura M (2011). Influenza vaccination of schoolchildren and influenza outbreaks in a school. Clin Infect Dis.

[53] Yugami S. (The Maebashin Research Group for the Study of Influenza Epidemics). Influenza epidemics in the non-vaccinated area. Report C-010. Tokyo: Toyota Research Foundation (2C-018); 1987

[54] Hirota Y (2008). Ecological fallacy and skepticism about influenza vaccine efficacy in Japan: the Maebashi study. Vaccine.

[55] Hirota Y, Kaji M (2008). History of influenza vaccination programs in Japan. Vaccine.

[56] Taubenberger JK, Morens DM (2006). 1918 influenza: the mother of all pandemics. Emerg Infect Dis.

[57] Leroux-Roels I, Leroux-Roels G (2009). Current status and progress of prepandemic and pandemic influenza vaccine development. Expert Rev Vaccines.

[58] Claas EC, Osterhaus AD, van Beek R, De Jong JC, Rimmelzwaan GF, Senne DA (1998). Human influenza A H5N1 virus related to a highly pathogenic avian influenza virus. Lancet.

[59] Fidler DP, Gostin LO (2012). The WHO pandemic influenza preparedness framework: a milestone in global governance for health. JAMA.

[60] World Health Organization. Conference report: Report of the 6th meeting on the evaluation of pandemic influenza vaccines in clinical trials. World Health Organization, Geneva, Switzerland, 17–18 February 2010. Vaccine 2010;28:6811–6820 10.1016/j.vaccine.2010.07.034 20659520

[61] Nakayama T, Kashiwagi Y, Kawashima H, Kumagai T, Ishii KJ, Ihara T (2012). Alum-adjuvanted H5N1 whole virion inactivated vaccine (WIV) enhanced inflammatory cytokine productions. Vaccine.

[62] Kamiya H, Uehara S, Kato T, Shiraki K, Togashi T, Morishima T (1998). Childhood bacterial meningitis in Japan. Pediatr Infect Dis J.

[63] Sunakawa K, Nonoyama M, Takayama Y, Yamaguchi Y, Ooishi T, Iwata S, et al. The trend of childhood bacterial meningitis in Japan (1997.7–2000.6). Kansenshogaku Zasshi. 2001;75:931–939 (in Japanese). 10.11150/kansenshogakuzasshi1970.75.931 11768357

[64] Yoshioka K, Tsuzuki D, Hiyama Y, Nakayama T, Togashi T, Kamiya H. A post-marketing clinical study of Haemophilus influenza type b [Hib] conjugate vaccine (ActHIB^R^): interim analysis of primary immunization. Nihon Shounikagakukai Zazzhi 2011;115:570–577 (in Japanese).

[65] Chiba N, Morozumi M, Sunaoshi K, Takahashi S, Takano M, Komori T (2010). IPD Surveillance Study Group. Serotype and antibiotic resistance of isolates from patients with invasive pneumococcal disease in Japan. Epidemiol Infect.

[66] Chiba N, Morozumi M, Ubukata K. Morphological changes penicillin-resistant *Streptococcus pneumoniae* and beta-lactamase-nonproducing, ampicillin-resistant *Haemophilus influenzae* after exposure to oral antibacterial agents. Jpn J Antibiot 2012;65:323–334. 23383434

[67] Sasaki K, Higashihara M, Inoue K, Igarashi Y (1976). Studies on the development of a live attenuated mumps virus vaccine. Kitasato Arch Exp Med.

[68] Hoshino M, Nishimitsu M, Ichimori Y, Oka Y, Kouno R, Yamashita K, et al. Development of live attenuated mumps Torii vaccine strain: development and biological characteristics. Clin Virol 1981;9:323–330 (in Japanese).

[69] Nagai T, Okafuji T, Miyazaki C, Ito Y, Kamada M, Kumagai T (2007). A comparative study of the incidence of aseptic meningitis in symptomatic natural mumps patients and monovalent mumps vaccine recipients in Japan. Vaccine.

[70] Plotkin SA, Rubin SA. Mumps vaccine. In: Plotkin SA, Orenstein W, Offit P (eds) Vaccines, 5th edn. Philadelphia: Saunders Elsevier, 2008. p 435–465.

[71] Hashimoto H, Fujioka M, Kinumaki H (2009). Kinki Ambulatory Pediatrics Study Group. An office-based prospective study of deafness in mumps. Pediatr Infect Dis J.

[72] Takahashi M, Asano Y, Kamiya H, Baba K, Ozaki T, Otsuka T, Yamanishi K (2008). Development of varicella vaccine. J Infect Dis.

[73] Izawa T, Ihara T, Hattori A (1977). Application of a live varicella vaccine in children with acute leukemia or other malignant diseases. Pediatrics.

[74] Asano Y, Nakayama H, Yazaki T (1977). Protection against varicella in family contacts by immediate inoculation with live varicella vaccine. Pediatrics.

[75] Ozaki T (2013). Varicella vaccination in Japan: necessity of implementing a routine vaccination program. J Infect Chemother.

[76] Kawabe Y, Sugiyama K, Wada Y, Yamada K (1989). 3-year study for the prevention of perinatal HBV infection under the standard method of the Ministry of Health and Welfare, Japan. Acta Paediatr Jpn.

[77] Komatsu H, Inui A, Sogo T, Hiejima E, Kudo N, Fujisawa T (2009). Source of transmission in children with chronic hepatitis B infection after the implementation of a strategy for prevention in those at high risk. Hepatol Res.

